# Tuberculosis and anemia—cause or effect?

**DOI:** 10.1186/s12199-021-01013-4

**Published:** 2021-09-21

**Authors:** Frank Cobelens, Andrew D. Kerkhoff

**Affiliations:** 1 Department of Global Health and Amsterdam Institute for Global Health and Development, Amsterdam University Medical Centers, location Academic Medical Center, Amsterdam, Netherlands; 2 Division of HIV, Infectious Diseases and Global Medicine, Zuckerberg San Francisco General Hospital and Trauma Center, University of California San Francisco, CA San Francisco, USA

We read with interest the systematic review by Gelaw et al. [1] from which the authors conclude that anemia is a risk factor for tuberculosis (TB), i.e., anemia precedes the development of TB disease. They explain this finding by immunosuppressive effects of anemia, either directly in the context of iron deficiency or indirectly through association with known risk factors for TB such as malnutrition, and recommend early diagnosis and treatment of anemia to help reduce the burden of TB. We believe that this conclusion is based on misinterpretation of the data.

In their meta-analysis of cross-sectional and case-control studies, the pooled odds ratio for the association between anemia and TB was 3.56 and increased with the severity of anemia. However, the meta-analysis of cohort studies showed a pooled hazard ratio of TB among anemic versus non-anemic participants of only 2.01, with no significant difference between mild, moderate, and severe anemia. This discrepancy suggests that the association between anemia and TB is the reverse, i.e., TB being a risk factor for anemia such that TB disease precedes the development of anemia.

This suggestion is further strengthened by the timepoint in some of the cohort studies for which the anemia data were used in the meta-analysis. From our cohort study of HIV-infected patients in South Africa they used the baseline anemia severity at antiretroviral treatment (ART) initiation, which showed only a weak, non-significant association with subsequent development of TB (rate ratios 0.96, 1.27, and 1.42 for mild, moderate, and severe anemia, respectively) [2]. However, our paper also presented time-updated anemia classifications during cohort follow-up, which were strongly predictive of TB occurring in the next 4 months (rate ratios 2.15, 5.01, and 13.61, respectively). Three other cohort studies included in Gelaw et al.’s meta-analysis presented time-updated anemia data and found time-updated anemia to be independently and highly predictive for incident TB, including shortly after ART initiation when these studies found high TB incidences [3–5]. Notably, each study also demonstrated a dose-response relationship between time-updated anemia severity and incident TB over periods up to 6 months. In other words, anemia predicts TB primarily over short periods of time.

The likely explanation for this finding is that anemia is an early marker of TB pathology that develops in the months before clinical TB disease becomes apparent. This is in line with findings of other biomarkers of inflammation already being detectable during that period [6]. Indeed, several studies have previously found a majority of TB patients to have hematologic and inflammatory profiles consistent with anemia of chronic disease [7, 8]. Mechanistically, hepcidin may be an important mediator of early TB-associated anemia; hepcidin concentrations in TB patients have been shown to be strongly and positively associated with mycobacterial burden, and are also strongly correlated with more severe anemia early during TB pathology [9]. Further evidence that TB disease likely drives the development of anemia, rather than the other way around, is the observation that anemia generally resolves following TB therapy [7, 10].

While we argue that Gelaw et al.’s meta-analyses do not support that anemia is an upstream risk factor for TB, their concluding recommendation is nonetheless valid: in TB high-incidence settings it may be useful to screen for anemia, as it may help detect and treat TB in an early stage, and thereby reduce morbidity and transmission.

## Data Availability

Not applicable.
